# Epigenetic aging in cows is accelerated by milk production

**DOI:** 10.1080/15592294.2023.2240188

**Published:** 2023-08-02

**Authors:** Prashansa Ratan, Liudmilla Rubbi, Michael Thompson, Kaushik Naresh, Jolena Waddell, Barbara Jones, Matteo Pellegrini

**Affiliations:** a Molecular, Cell and Developmental Biology, University of California Los Angeles, Los Angeles, CA, USA; b Department of Zoology, Dayalbagh Educational Institute (Deemed to Be University), Dayalbagh, Agra, UP, India; c Department of Mathematics, University of California Los Angeles, Los Angeles, CA, USA; d Department of Animal Science, Tarleton State University, Stephenville, TX, USA; e Texas A&M AgriLife Research, Stephenville, TX, USA

**Keywords:** Epigenetic clock, epigenetic pacemaker, cows, bovine, DNA methylation

## Abstract

DNA methylation has proven to be the most promising age-predictive biomarker in mammals resulting in the emergence of ‘epigenetic clocks’ that describe the relationship between methylation levels and age. Using Targeted bisulfite Sequencing, we evaluated blood DNA-methylation data from 96 domesticated cows (*Bos Taurus*) of which 88 were adults and 8 were calves. This allowed us to measure DNA methylation across three thousand regions in the genome that were conserved across mammals. The significant association of age with the changes in DNA methylation enabled us to construct an epigenetic clock that predicts the age of cows to within nine months. We also investigated whether factors exist that moderate the association between epigenetic age and actual age and found that milk production levels significantly increase the rate of epigenetic ageing, suggesting that the stress of excessive milk production might be accelerating epigenetic ageing in cows.

## Introduction

The genome of the domesticated cow, *Bos taurus*, was sequenced and annotated in 2009. The size of the bovine genome is approximately 3 billion base pairs, which is similar to the size of the genomes of humans and other mammals. Although humans and primates are phylogenetically distant from the Artiodactyla, which includes the domesticated cow, they share a large percentage of their genes. Analysis of the bovine genome revealed that out of 18,019 human genes, 17253 genes (95.7%) had significant homologs in cows [1,2]. The study of cows has informed our understanding of fertility in women, due to the similarity with human physiology related to follicle selection, and gestation period among other traits [3]. Recently, a web portal known as CattleGTEx atlas has been made available to the public and serves as a primary reference for cattle genomics, breeding, adaptive evolution, veterinary medicine and comparative genomics [4]. Interestingly, the most represented breed in the CattleGTEx portal is the Holstein cow (35.5% of all samples).

Milk is a valuable commercial commodity and with the help of improved genetics, selection, and management, the production of milk by the modern dairy cow exceeds the amount required to feed the offspring. The primary factor limiting milk production in cows is the number of milk-synthesizing cells in the mammary gland [5,6]. The mammary gland of dairy cattle undergoes three cycles of development, lactation, and involution. The mammary epithelial cells (MEC) synthesize milk fat, milk protein [7] and lactose using metabolites from the blood [8]. Studies also suggest that *DOCK1, PTK2, and PIK3R1* are important genes associated with milk production traits in dairy cattle [9].

The world’s most productive dairy animal, the Holstein cow, is the result of comprehensive genetic and breeding programmes to augment milk productivity [10,11]. Breeding programmes have developed Holstein cows that can produce 10,000 kg of milk/year, which converts to more than 33 kg/day [12,13]. Therefore, Holstein cows have become an excellent model system for studying the impact of high milk production on physiology. Several differentially expressed genes (DEGs) in the liver have been identified during the three lactation periods: dry period (50-d prepartum), early period (10-d postpartum) and peak of lactation (60-d postpartum) [14]. These include APOC2, PPP1R3B, PKLR, ODC1, DUSP1, LMNA, GALE, ANGPTL4, LPIN1 and CDKN1A, and may affect milk production traits such as milk yield, fat traits and milk protein in dairy cattle [14]. Molecular pathways involving cytokine-activated Janus kinase (JAK) and signal transducer and activator of transcription (STAT) have also been show to impact milk production [15].

While many studies have examined the genetic basis of cow traits, not many have investigated cow epigenetics. Unlike genetics, epigenetic changes are reversible and do not change the DNA sequence but alter gene expression in a cell type specific manner. Alterations in DNA methylation also lead to epigenetic drift which can occur with age. Studies have shown that DNA methylation plays a regulatory role in gene transcription, resulting in the modification of milk protein gene expression [16] which in turn affects milk production. It has also been observed that DNA methylation plays an integral role in regulating the expression of important milk protein genes in the mammary gland during lactation both in mouse [17] and in dairy cattle [18]. Epigenetic mechanisms may also play a significant role in modulating other factors that influence cell number and milk production, which include, but are not limited to, farm management practices such as nutrition, pregnancy, milking frequency, photoperiod, and even diseases like mastitis, milk fever, etc.

A cow has a natural lifespan of 15–20 years. However, the lifespan is often shortened to 4 to 6 years due to dairy and/or beef practices. Previous studies have shown that DNA methylation profiles in cattle are influenced by age [19–21]. It has been reported that in many tissues of diverse organisms, from salmon, cattle, rats and mice to humans, the overall level of DNA methylation decreases with age [22–27]. In several mammals there is a nonlinear relationship between DNAm levels and animal age, with the rate of changes in methylation decreasing with age [21].

Several prior studies have investigated the development of epigenetic clocks for cows. An epigenetic clock was constructed to measure the age of oocytes using the HorvathMammalian40K array, which contains 37,000 mammalian CpGs sites [3]. Another epigenetic clock for tropically adapted cattle was derived from tail hair (a tissue widely used in industry for genotyping) and using portable sequencing devices [28]. Finally, an epigenetic clock for cattle (*Bos taurus*) was constructed using the custom mammalian methylation array ‘HorvathMammalMethyl40K’ from TSU (ear tissue punches) samples, showing high accuracies to the individual species’ clocks (*r* > 0.97) and utilizing only 217 CpG sites to estimate age [29].

To study the dynamics of DNA methylation, we developed quantitative models that measure changes in DNA methylation with age as well as the effect of multiple factors on DNA methylomes, including milk production, reproductive status, number of lactations and days carried calf. We collected bovine blood samples from 96 Holstein cows and used targeted bisulfite sequencing to measure methylomes at approximately three thousand loci. We investigated the relationship between DNA methylation and age, along with other factors.

## Materials and methods

### Bovine samples

**Figure 1.**
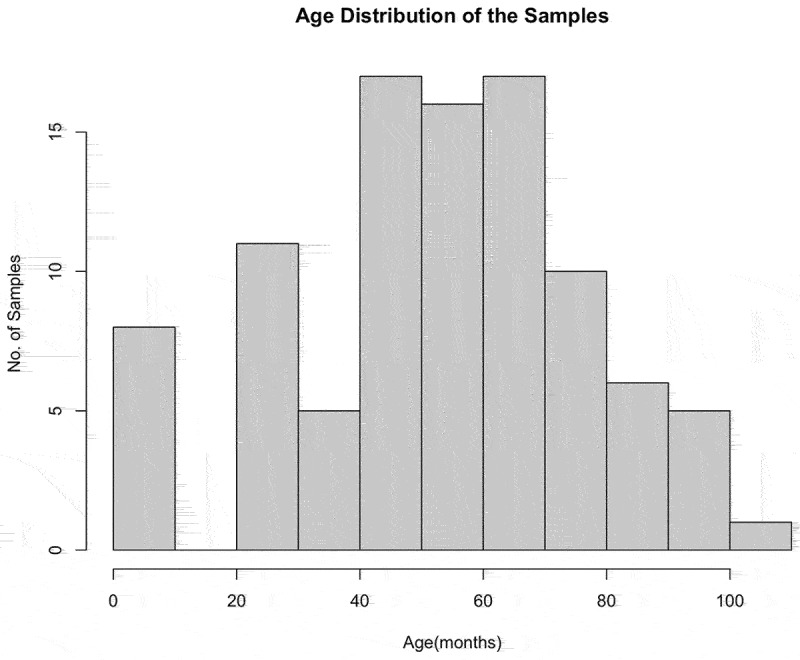
Histogram of Age Distribution of Samples. Age distribution of cow samples used in the study.

Mature crossbred lactating cows (*n* = 93) were housed at the Southwest Regional Dairy Center (Tarleton State University, Stephenville, Texas) and sampled under Animal Care and Use Protocol 10-021-2018. Calves (4-month-old heifers, *n* = 8) were owned and sampled by a private producer in Central Texas and blood samples were graciously donated to the study. The hybrid cattle used in the study were Holstein X Jersey crossbred dairy cows. All animals were bled by coccygeal venipuncture into 10 mL lavender K2EDTA Vacutainers (Becton, Dickinson and Company, Franklin Lakes, New Jersey). Blood was stored at 4°C until DNA extraction was performed using 300 µL of blood in the fresh blood protocol of the Genomic DNA Mini Kit (IBI Scientific, Dubuque, Iowa). Genomic DNA was eluted in 100 µL of elution buffer, quantified by Qubit and stored at −20°C until further analysis. The age distribution of the samples is described through the histogram (Figure 1).

All the bovine traits are described in Supplementary Table S3.

### Targeted bisulfite sequencing

We applied targeted bisulfite sequencing (TBS-seq) to characterize the methylomes of 96 DNA cow samples. The protocol is described in detail in [31]. Briefly, 500 ng of genomic DNA were used for TBS-seq library preparation. Fragmented DNA was subject to end repair, dA-tailing and adapter ligation using the NEBNext Ultra II Library prep kit (NEB) and custom pre-methylated dual unique index adapters (IDT). Pools of 16 purified libraries were hybridized to 3572 biotinylated probes specific for conserved sequences in mammals (IDT). The sequence of the probes used in this study can be found in Supplementary Table S1.

The Hybridization was carried out using the xGen hybridization capture kit (IDT) according to the manufacturer’s instructions. Captured DNA was bisulfite treated with the Zymo Gold kit (Zymo Research) prior to PCR amplification using KAPA HiFi Uracil+(Roche). The following conditions were used for the PCR amplification: 2 min at 98°C; 14 cycles of (98°C for 20 sec; 60°C for 30 sec; 72°C for 30 sec); 72°C for 5 minutes; hold at 4°C. Library QC was performed using the High-Sensitivity D1000 Assay on a 4200 Agilent TapeStation. Pools of 96 libraries were sequenced on a NovaSeq6000 (Sp lane) as paired-end 150 bases.

### Data processing

Demultiplexed fastq files were aligned to the bovine genome ARS-UCD1.2/bosTau9 using BSBolt Align (v1.3.0) [30]. Before calling methylation using BSBolt CallMethylation function, PCR duplicates were removed using samtools markdup function (samtools version 1.15). CGmap files were generated to describe the methylation status of the observed cytosines using the BSBolt CallMethylation function. These CGmap files are assembled into a consensus methylation matrix using the function BSBolt AggregrateMatrix. The minimum site read depth coverage of 10 and a minimum coverage threshold of 0.8 required for the proportion of samples that must have a valid site was used to build the aggregate matrix [30]. The resulting methylation matrix had 8408 methylation sites (Supplementary Table S2).

### Epigenetic clock

The package glmnet was used for building the penalized regression models [31]. In order to optimize the input of the number of predictors of CpGs, we utilized the ‘elastic net’ version of glmnet corresponding to the alpha parameter of 0.5. Internal cross-validation (cv.glmnet) was employed to automatically select the optimal penalty parameter. Leave-one-out cross-validation (LOOCV) training method was used to predict the age of individual cows, wherein each predicted cow represented the testing set and the rest was the training set against which the age of an individual was predicted.

### Moderation analysis

To identify the factors that moderate the relationship between the actual age and the predicted age we used linear models and computed the p-value for each term using Linear Regression and lm() function in R. We used 96 cows to train the model. The moderators we tested were milk production, reproductive status (0 = calf, 1 = preg, 2 = bred, 3 = OK/open, 4 = fresh), days carried calf and number of lactations. The significance of each term was calculated with the matrix of factors input as the independent variables and the epigenetic age predictions as the dependent variable.

### Epigenetic pacemaker

The Python package EpigeneticPacemaker (EpigeneticPacemaker.EpigeneticPacemaker) was used to generate predictions for each cow’s epigenetic state [32]. We used the Pearson correlation coefficient to select the top 8408 methylation sites that were highly correlated with age. The minimum correlation threshold was set to be 0.5.

#### EWAS

Epigenome Wide Association Analysis was performed using the R package ‘qqman.’ We display the results of this analysis using Manhattan and Q-Q plots. In this study, age, milk production, number of lactations, reproductive status and days carried calf were the phenotype of interest and the association score is calculated as –*log*10(*P*-value) on the y-axis versus the chromosomal position of the CpG site on the x-axis. The two horizontal lines in the Manhattan plot are the suggestive line and genome-wide line respectively, based on the chromosome-wide or genome-wide Bonferroni threshold. We used the Benjamini-Hochberg procedure to correct the *P* values for multiple testing. The closest gene to each CpG site was found in the bovine genome (ARS-UCD1.2/bosTau9) using the UCSC genome browser.

## Results

### Bovine methylomes

We collected DNA from 96 bovine blood samples, of which 88 were adults and 8 were calves. The age of the bovine samples ranged from 4 months to 8 years and 9 months. Targeted bisulfite sequencing libraries were prepared from these samples. DNA was sheared, and libraries were prepared using premethylated adapters. We then carried out targeted enrichment using a panel of 3387 probes designed to hybridize to the regions of the genome that are conserved across mammals (Supplementary Table S1). Following bisulfite conversion, the final library pools were sequenced on an Illumina Novaseq. The resulting reads were then aligned to an index of the cow genome using BSBolt. The aligned reads were used to generate methylation matrices that measured the methylation of captured regions across the samples. Details of the samples, library preparation, and data analysis are provided in the Methods section.

### Age associated changes in DNA methylation

**Figure 2.**
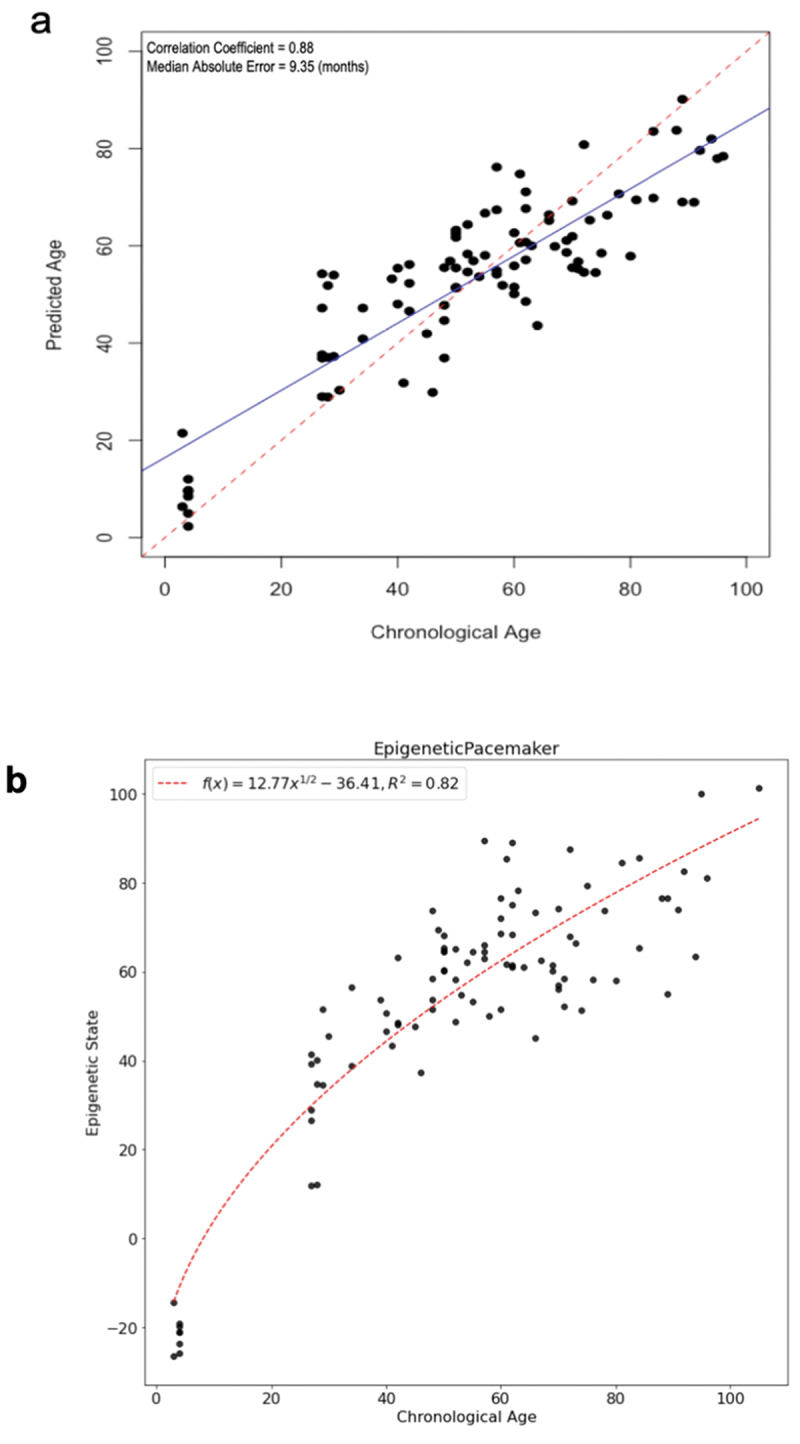
Epigenetic age and state of cows. (a) Models were generated using elastic net regression. (b). The Epigenetic Pacemaker was used to predict epigenetic states of the bovine samples. The trend line was fit using a non-linear function.

Two complementary approaches were used to study DNA methylation changes associated with age: the epigenetic clock and epigenetic pacemaker. Epigenetic clocks are an efficient and reliable method to predict the age of an animal based on their methylation profile. DNA methylation clocks, or epigenetic clocks, are generally built using supervised machine learning methods such as penalized regression. Here we used elastic net regression as implemented in the glmnet R package. The methylation data used to train the model consisted of eight thousand four hundred and eight CpG sites that were covered with at least 10 reads across all of our samples. To avoid overfitting, we used leave-one-out cross-validation to build an individual model for each sample which was trained on all the remaining data excluding the test sample. Our resulting model is able to predict the age of cows to within an average absolute error of approximately nine months (Figure 2a), although we observe that these models tend to under-predict the age of calves, and therefore we also explored alternative approaches to model the changes in methylation with age. The epigenetic clock we developed using targeted bisulfite sequencing compares favourably to two previously published clocks based on correlation and mean absolute error between predicted and actual ages (see Table 1).

DNA methylation changes associated with age are often non-linear with time, with faster rates of change early in life that decrease with age. To test whether this is occurring in our samples, we have previously developed the epigenetic pacemaker. This approach is complementary to the regression based approach used in epigenetic clocks. The epigenetic pacemaker (EPM) is a linear model of DNA methylation values with respect to an unknown variable we refer to as the epigenetic state:

$$m_{ij}=\;{m_{i}}^{0}+\;r_{i}s_{j}$$

wherein, *i* is the CpG site and *j* the individual, *m*_*ij*_ represents the methylation level of position *i* in individual *j*, *m*^*0*^ represents the methylation level at birth (i.e., the initial methylation values), *r*_*i*_ is the rate of change and *s*_*j*_ is the epigenetic state. The epigenetic state of each individual represents a position in the epigenetic trajectory of its lifespan, and we do not assume a priori that the epigenetic state changes linearly with time, but rather allow the optimization to identify this relationship in an unbiased fashion. The underlying working of the EPM algorithm is a fast conditional expectation maximization (EM) algorithm wherein each methylation site is assigned an independent rate of change (*r*_*i*_), an initial methylation value (*m*_*i*_^*o*^) and each individual is assigned an epigenetic state (*s*_*j*_) that is initially set to the actual age of the sample. The EM process is repeated until the model converges and minimizes the difference between the observed and predicted methylation values in our dataset.

Previous studies have shown that in humans the logarithmic function provides a good fit for the association of epigenetic age with actual age. The same was also shown in dogs [8]. We find that in our cow samples the relationship between epigenetic state and actual age is well fit by a square root function (Figure 2b). These results are consistent with those we have observed in humans and dogs and suggest that the rate of DNA methylation change is decreasing as the cow age increases.

### EWAS analysis

**Figure 3.**
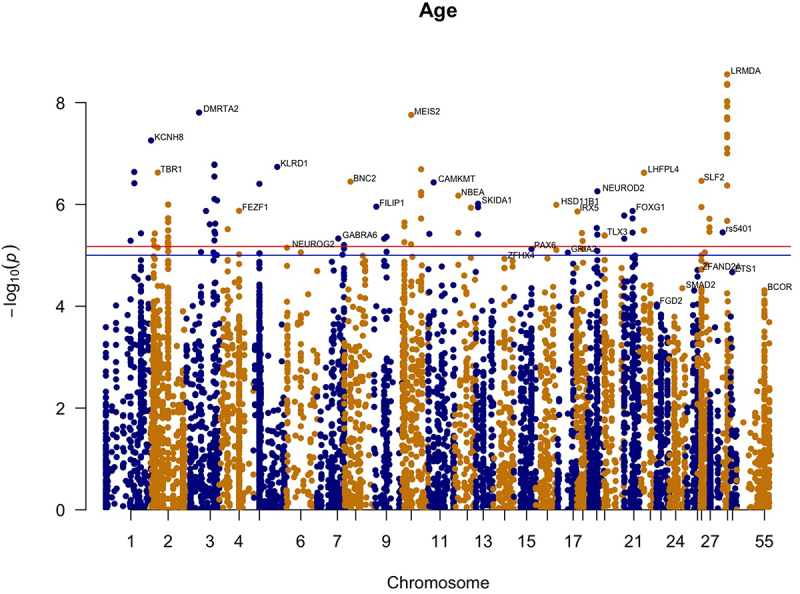
**Epigenome-wide association results for age**. Manhattan plot representing epigenome-wide association results for age. 8408 CpG sites were included. CpG sites are plotted on the x-axis ordered by position and the y-axis shows the -log_10_(p) of the association.

To identify individual methylation sites that show age associated change, we performed Epigenome Wide Association Analysis on 8408 CpG sites. In order to measure the relationship between the methylation and age, we used the lm() function in R to calculate the p-values and the correlation coefficient between SNPs and methylation sites. We used the Bonferroni procedure to find the significantly correlated sites. The closest gene to each site was found using the Ensembl browser (ARS-UCD1.2). The top age-related genes are as follows (Figure 3 and supplementary figure S1): K*CNH8, TBR1, DMRTA2, FEZF1, KLRD1, NEUROG2, GABRA6, BNC2, FILIP1, MEIS2, CAMKMT, NBEA, SKIDA1, ZFHX4, PAX6, GRIA2, HSD11B1, IRX5, TLX3, NEUROD2, FOXG1, LHFPL4, SLF2, FGD2, SMAD2, ZFAND2A, ETS1, BCOR, LRMDA* (Table 2).

**Table 1.** Bovine Epigenetic clocks.

Clock	Tissue	Sample Size (n)	Correlation Coefficient (r)	Mean Absolute Error (MAE) (months)	Assay
Kordowitzki et al., 2021	Blood-Oocyte	357	0.9	8.79	Methylation array (40K)
Kordowitzki et al., 2021	Blood	277	0.91	8.86	Methylation array (40K)
Hayes et al., 2021	Tail hair	66	0.71	16 (*n*<3yrs) 17 (*n*=3-10yrs)	Oxford nanopore (37K)
Our study	Blood	96	0.88	9.35	Targeted bisulfite sequencing(8K)

**Table 2.** Top Significant genes for Age.

Chromosome site	Chromosme number	Closest gene from site	Strand	Distance to gene (bp)
155797851	1	KCNH8	1	501282
34966494	2	TBR1	−1	9902
45847764	3	DMRTA2	1	5079
68801404	4	FEZF1	−1	3006
87471453	5	KLRD1	−1	4777
13061269	6	NEUROG2	1	1492
73272273	7	GABRA6	1	16788
27513731	8	BNC2	1	475936
14932363	9	FILIP1	−1	218085
32619863	10	MEIS2	−1	223131
27195648	11	CAMKMT	1	427048
25982827	12	NBEA	−1	671846
22724269	13	SKIDA1	−1	2768
39849062	14	ZFHX4	1	202050
62569850	15	PAX6	−1	27906
73609682	16	HSD11B1	−1	69590
41789795	17	GRIA2	−1	186515
23238778	18	IRX5	1	5989
39988762	19	NEUROD2	−1	4124
3160122	20	TLX3	1	2652
39241904	21	FOXG1	1	1472
17088486	22	LHFPL4	1	31434
9191745	23	FGD2	1	26289
47035393	24	SMAD2	−1	82882
41617913	25	ZFAND2A	1	8040
21719705	26	SLF2	1	39509
31139646	28	LRMDA	1	26038
29998660	29	ETS1	−1	136647
103336063	55	BCOR	1	23734

We performed functional enrichment analysis of these genes using the EnrichR tool [33–36] (Table 3). We have seen in previous studies that CpG sites that change with age are often associated with polycomb repressive complex binding sites (PRC) [37]. For example, JARID2, SUZ12 and EZH2 are transcription factors associated PRC and with the H3K27 trimethylation mark. Another factor we identified is REST, which is associated with the suppression of neural specific genes. This factor has been observed in other studies of age associated DNA methylation changes [38].

**Table 3.** Age.

*Term*	*Overlap*	*P-value*	*Adjusted* *P- value*
*REST CHEA*	7/1280	.001940325	.033726991
*REST ENCODE*	4/383	.002152787	.033726991
*REST 21,632,747 ChIP-Seq MESCs Mouse*	9/1765	6.24E–04	.009608245
*H3K27me3 Stomach Smooth Muscle*	7/993	4.34E–04	.126809376
*H3K27me3 CD8 Naive Primary Cells*	10/2258	8.70E–04	.126996471

We also carried out an EWAS analysis between methylation and different phenotypic traits including milk production, reproductive status, number of lactations and days carried calf (Supplementary Figures S3-S5). Table 4 shows the number of samples associated with each trait that was considered for the EWAS analysis.

The ‘milk yield’ phenotype data used in the EWAS analysis is the cow’s milk yield in pounds on a daily basis. The milk yield information was recorded on one day in the month prior to sampling and recorded each cow’s milk yield for that day.

**Figure 4.**
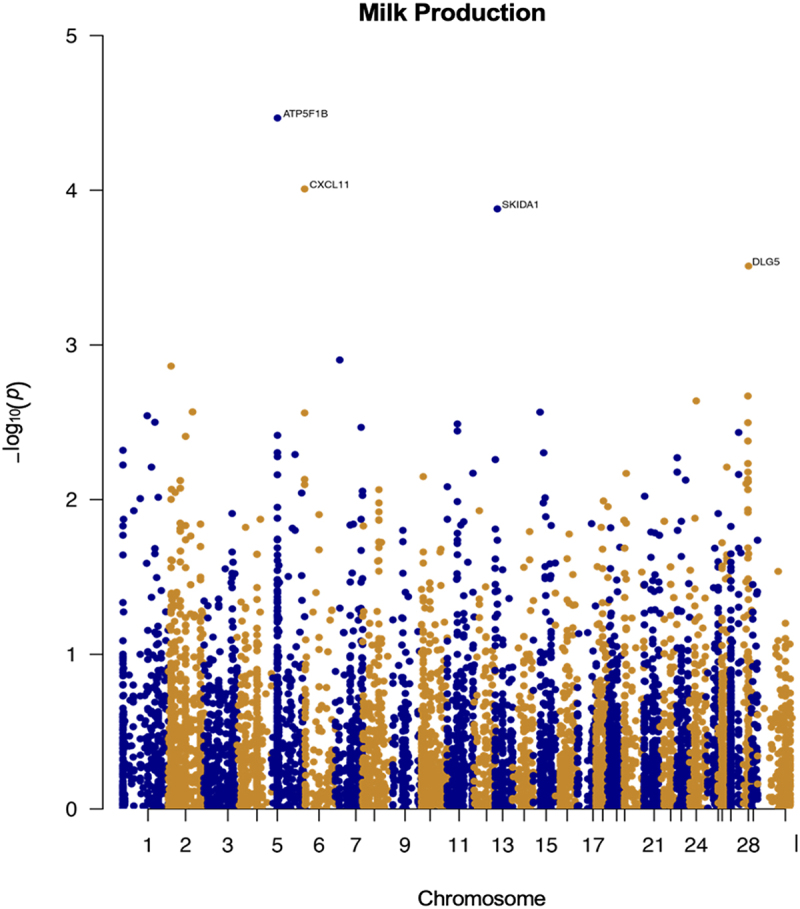
**Epigenome-wide association results for milk production**. Manhattan plot representing epigenome-wide association results for milk production. CpG sites are shown on the x-axis ordered by position and the y-axis shows the -log_10_(p) for the association.

Following the same procedure described for age associated change, and the top milk production-related genes are (Figure 4 and supplementary figure S2): *ATP5F1B, CXCL11, SKIDA1* and *DLG5* (Table 5). However, unlike the age associated sites, none of these reached the Bonferroni threshold, and are therefore only suggestive of a possible association that will need to be confirmed with larger sample sizes.

**Table 4.** Number of samples for each trait.

Phenotypes	No. of samples
REPRODUCTIVE STATUS	87
PREG	37
BRED	38
FRESH	10
OK/OPEN	2
NO. OF LACTATIONS	87
FIRST LACTATION	11
SECOND LACTATION	11
THIRD LACTATION	24
FOURTH LACTATION	22
FIFTH LACTATION	10
SIXTH LACTATION	6
SEVENTH LACTATION	3
DAYS CARRIED CALF	87
RANGE	0–172

We again performed functional enrichment analysis of these genes using the EnrichR tool [34–36]. Our hypothesis is that excess milk production is associated with stress and higher production of milk leads to inflammation which is supported by the functional enrichment annotation of milk production associated sites which show enrichment for IL6, Interferon alpha and TNF alpha, all of which as associated with inflammation [39] (Table 6).

**Table 5.** Top Significant genes for Milk Production.

Chromosome site	Chromosme number	Closest gene from site	Strand	Distance to gene (bp)
26310461	5	ATP5F1B	1	30479034
11101140	6	CXCL11	−1	79801580
22724269	13	SKIDA1	−1	4088
33387233	28	DLG5	−1	27111

**Table 6.** Milk Production.

*Term*	*Overlap*	*P-value*	*Adjusted* *P- value*
IL-6/JAK/STAT3 Signaling	1/1987	.01728794	.03940669
Interferon Alpha Response	1/1987	.01926061	.03940669
TNF-alpha Signaling via NF-kB	1/200	.03940669	.03940669
Oxidative Phosphorylation	1/200	.03940669	.03940669
Interferon Gamma Response	1/200	.03940669	.03940669
Estrogen Response Late	1/200	.03940669	.03940669
Inflammatory Response	1/200	.03940669	.03940669

### Moderation analysis

**Figure 5.**
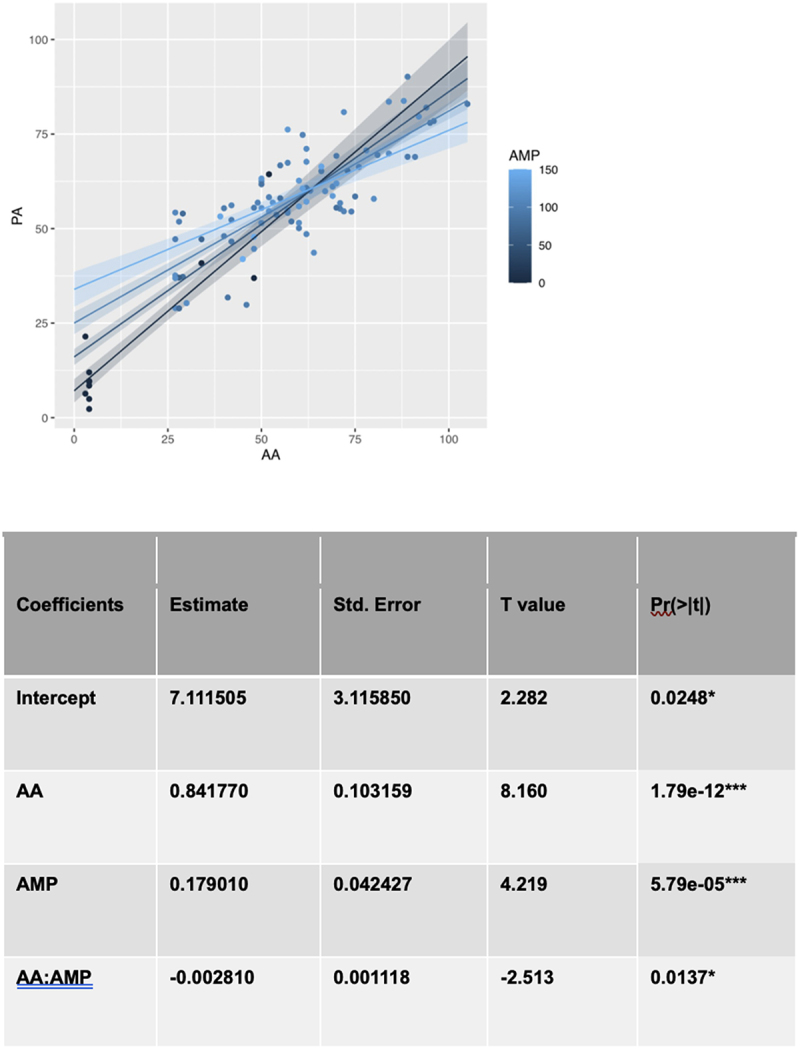
Moderation Analysis. a regression model of predicted age using three variables: Actual Age(AA), Actual Milk Production(AMP) and the product of actual age and actual milk production(AA*AMP). Regression lines are shown for different levels of milk production.

To identify the factors that moderate the relationship between the actual age and the predicted age we used multiple linear regressions and computed the p-value for each term to assess whether any other factor is associated with age acceleration. Multiple factors were tested for significant moderation across the 96 cows in our dataset. These included milk production, reproductive status (0 = calf, 1 = preg (confirmed pregnancy), 2 = bred (cows have been bred but not confirmed pregnant), 3 = OK/open (not pregnant), 4 = fresh (recently calved and just started milking)), days carried calf and number of lactations. The significance of each term was calculated by modelling the predicted age using the actual age, the factor and the product of the factor with age. The significance of each factor was measured by the *P* values associated with the factor and product term in each model. We found that among all of the factors we tested, only milk production was a significant moderator. In this model the coefficient for milk production is significant and positive while the interaction term is only significant at the 5% level and negative. This suggests that milk production accelerates epigenetic ageing for cows but does so in a manner that decreases with age (Figure 5).

## Discussion

We aimed to develop epigenetic clocks for cows that are based on DNA methylation patterns measured in blood. Using blood samples from cows of known age, two approaches were used to model epigenetic ageing: the epigenetic clock (EC) and the epigenetic pacemaker (EPM). One of our objectives was to predict the age of an individual by using a weighted linear model of methylation sites. The epigenetic clock developed from blood samples to predict the chronological age predicted the age of an individual with a 9-month average error. Our study also demonstrates that similarly to humans and other mammals, cows have rapid changes in DNA methylation early in life that slow down with age. We also identified the significant genes that are strongly associated with age and milk production.

By modelling the time dependent changes in DNA methylation, the EPM model allows us to measure non-linear trends in DNA methylation across the lifespan of cows. Non-linear epigenetic ageing trends have been seen in humans and other species [40,41], suggesting that DNA methylation changes are rapid early in life and slow as organisms age. Along with the slowing of epigenetic changes we also observed an increased variability in epigenetic age in adult cows compared to calves. Similar trends of decreased variance with age have also been observed in human studies [42].

Among the traits we investigated, age and milk production were the phenotypes with the strongest association with DNA methylation. By carrying out association studies, we identified the genes closest to the significant CpGs. The top significant genes that were identified for the milk production were *ATP5F1B, CXCL11, SKIDA1*, and *DLG5*. The CXC chemokine family consists of two main clusters of chemokines, the Gro cluster and the IP-10 cluster as well as other non-cluster chemokines. Both the clusters are found on chromosome 4 in humans and chromosome 6 in cattle. The IP-10 cluster comprises three chemokines – CXCL9, CXCL10 and CXCL11. Cattle possess three genes that appear to be the direct homologues of *CXCL9, CXCL10*, and *CXCL11* in other species when compared phylogenetically [43]. These chemokines play an essential role in the permeation or accumulation of the immune cells in inflammatory lesion and studies have shown that IL-27 could induce CXCL11 production along with CXCL9 and CXCL10 in TR146, a human oral epithelial cell line that is supplemented with 10% foetal bovine serum, which implies that IL-27 could be involved in Th1 cells accumulation [44]. This result might suggest that high levels of milk production may induce inflammatory responses. Studies of DLG5 have shown that single nucleotide polymorphisms (SNP) associated with DLG5 are closely related to the reproductive traits in buffaloes [45], also linking this gene to milk production. It has been shown that ATP5F1B is present as one of the 543 milk fat globule membrane (MFGM) proteins that have been identified on goat colostrum and all these identified MFGM proteins in the colostrum and mature milk were mainly involved in 32 KEGG pathways [46]. Finally, studies have shown that *SKIDA1* among many others is one of the significantly regulated genes by colostrum exosome capsulated oligosaccharides in macrophages, which are responsible for the establishment of intestinal immunity [47]. It has also been seen that the expression of*SKIDA1* in the house mouse foetal heart increases, then decreases with age [48].

We found some association with ageing for 7 of the 29 age associated genes. Some of the genes that we found to be associated with age include *TLX3, FOXG1, KCNH8, TBR1, DMRTA2, FEZF1*, and *SKIDA1*. *TLX3* or T Cell Leukaemia Homeobox 3 encodes a DNA-binding nuclear transcription factor. The transcription factors have been considered important in the regulation of the genes which confer various biological functions associated with maturity and ageing [28]. FOXG1 plays a critical role in the auditory degeneration process through regulation of macroautophagy/autophagy [47]. *KCNH8* is a member of the human ElK K^+^ channel gene family. Voltage-gated potassium channels represent the most complex class of voltage-gated ion channels from both structural and functional standpoints. Their diverse functions include but are not limited to regulating neurotransmitter release, insulin secretion, heart rate, neuronal excitability, epithelial electrolyte transport and smooth muscle contraction [49]. *TBR1* belongs to a conserved family of genes that share a common DNA-binding domain, the T-box. T-box genes encode transcription factors involved in the regulation of numerous developmental processes. In mice, the ortholog of this gene is expressed in the cerebral cortex, hippocampus, amygdala and olfactory bulb and plays an important role in the neuronal migration and axonal projection. Studies have shown that the TBR1 CpG site demonstrates a strong and statistically robust linear relationship between DNA methylation and age in humans [50]. DMRTA2 is required for early embryonic development of the cerebral cortex in mice [51]. The Fez family zinc finger protein 1 FEZF1 is a C2H2 zinc finger transcription factor in nervous system development. It plays a critical role during forebrain and olfactory system development in vertebrates. FEZF1 promotes cell proliferation and migration by acting as a transcriptional activator of the Wnt signalling pathway and thereby plays an oncogenic role in cervical cancer [52]. *KLRD1* along with *KLR*, *KLRC3*, and *KLRG1* and many more have shown enhanced expression in NK cells. It is one of the most profound age-related changes in T cells, especially in human CD28^_^ CD8 T cells [53]. *NEUROG2* is a transcription factor gene that plays an important role in retinal neurogenesis. Expression of *NEUROG2* can be enhanced by SOX2. Finally, among the genes that showed a significant association between methylation and age, *SMAD2* may regulate the inverse relationship between the lifespan and the size of the adult dogs [54] and humans [55].

We recognize that our study has several limitations. Firstly, we only analysed blood samples, but there is also interest in the study of more easily collected tissues such as buccal swabs. Secondly, since the cows do not live their full natural lifespan, the age range for the samples represent only half of their natural lifespan. Absent the farming needs, the lifespan of cows could understate their longevity. The oldest living cow has been recorded as 48 years and nine months old. Considering that the beef and dairy industries are inseparable and 21% of the commercially sold meat are produced by dairy cows in 2019 just in the United States [56]. Interestingly, gender affects the calf’s longevity in agriculture. In the dairy sector, female calves are raised for meat if they are not able to produce enough milk and because of the unprofitable status, many male calves are killed as soon as they are born.

Our analysis of epigenetic ageing in cows extends prior studies in several ways. First, we have used a novel technology to probe DNA methylation that is cost effective and allows us to infer the age of cows by profiling only a couple of thousand locations in the genome. Compared to previously reported clocks, our clock had similar results with a correlation coefficient of *r* = 0.88 and mean absolute error of 9.35 months. The epigenetic clock with bovine blood samples generated a correlation coefficient of *r* = 0.91 and a mean absolute error of 8.86 months as reported by Kordowitzki et al., 2021. Similarly, a correlation coefficient of *r* = 0.71 and a mean absolute deviation of 16 months for animals aged less than 3 years of age, and 17 months for animals aged 3–10 years was reported by Hayes et al., 2021. Our clock has some advantages over the studies that have been conducted so far on the cattle epigenetic ageing. By measuring only a couple of thousand sites, our method is less expensive and more cost efficient compared to both methylation arrays and whole genome sequencing.

As we collected information other than age on the cows, we were also able to ask whether there was a significant association between epigenetics and milk production and whether the level of milk production influenced epigenetic ageing. Cows experience significant oxidative stress in early lactation as physiological pressure for milk synthesis results in an increase in energy and oxygen demand and also increases the production of reactive oxygen species (ROS) [57]. The act of giving birth (calving) and stopping milk production (dry-off) also causes stress in the dairy cow. Stressors assume a variety of forms creating strain, which affects multiple aspects of animal production from embryonic development to pregnancy outcome [58]. Studies have revealed that less strain in response to stress would make cows more fertile. Although there are multiple aspects of stress that affect cattle from heat and humidity, infectious disease, injury to milk production and undernutrition, the mediators of these stresses could be seen in the form of elevated body temperature, chronic pain, metabolic and hormonal imbalance, and inadequate nutrient intake to name a few. The final effect of these stresses could be seen in the embryonic development phase or even during pregnancy [58]. Studies have also shown that because of metabolic heat production associated with high milk production, lactating cows are particularly sensitive to heat stress. Also, a commonality between beef and dairy cattle is the environmental stress caused by heat and humidity (heat stress), although the strain associated with heat stress which is the elevated body temperature is greater in dairy cows [59,60].

It has been proven that there is a strong correlation between cow longevity and milk production levels wherein lower production cows live a longer life than higher production cows [29]. Our results are consistent with previous findings and we hypothesize from the results of our study that the stress of producing large quantities of milk accelerates the epigenetic ageing process in cows. This suggests that the breeding of cows for high levels of milk production may come at the expense of their longevity.

## Supplementary Material

Supplemental Material

## Data Availability

The authors confirm that the data supporting the findings of this study are available within the article and its supplementary materials https://drive.google.com/drive/folders/1HHmLP6ElTVcbjZw9bbdEQcP2ak2Fa3-x?usp=sharing.
