# Supplementary material for: Epigenetic aging in cows is accelerated by milk production
Source: Epigenetics. 2023 Aug 2;18(1):2240188. doi: 10.1080/15592294.2023.2240188 (PMC10402850; doi:10.1080/15592294.2023.2240188)
Supplement: Supplemental Material [file KEPI_A_2240188_SM7744.zip › Supplementary files/Supplementary captions.docx]

**Supplementary Figure 1. Q-Q plot for Age.** Expected versus observed values of the probability distribution. We observe an excess of extreme p-values.

**Supplementary Figure 2. Q-Q plot for Milk Production.** Expected versus observed values of the probability distribution.

**Supplementary Figure 3. Epigenome-wide association results for DCC.** Manhattan plot representing epigenome-wide association results for days carried calf. CpG sites are ordered on the x-axis as by chromosomal position and the y-axis shows the -log10(p) for the association. There were no significant associations.

**Supplementary Figure 4. Epigenome-wide association results for Number of Lactations.** Manhattan plot representing epigenome-wide association results for number of lactations. CpG sites are ordered on the x-axis as by chromosomal position and the y-axis shows the -log10(p) for the association. There were no significant associations.

**Supplementary Figure 5. Epigenome-wide association results for Reproductive Status.** Manhattan plot representing epigenome-wide association results for reproductive status. CpG sites are ordered on the x-axis as by chromosomal position and the y-axis shows the -log10(p) for the association. There were no significant associations.

**Supplementary Table 1. Sequence of the probes.** The sequence of the probes that were used in the study.

**Supplementary Table 2. Methylation matrix.** The methylation matrix with 8408 methylation sites.

**Supplementary Table 3. Phenotype Data.** The bovine traits that were used in the study.
