# Supplementary material for: Epigenetic aging in cows is accelerated by milk production
Source: Epigenetics. 2023 Aug 2;18(1):2240188. doi: 10.1080/15592294.2023.2240188 (PMC10402850; doi:10.1080/15592294.2023.2240188)
Supplement: Supplemental Material [file KEPI_A_2240188_SM7744.zip › Supplementary files/Supplementary Figures.docx]

**Supplementary Figures**


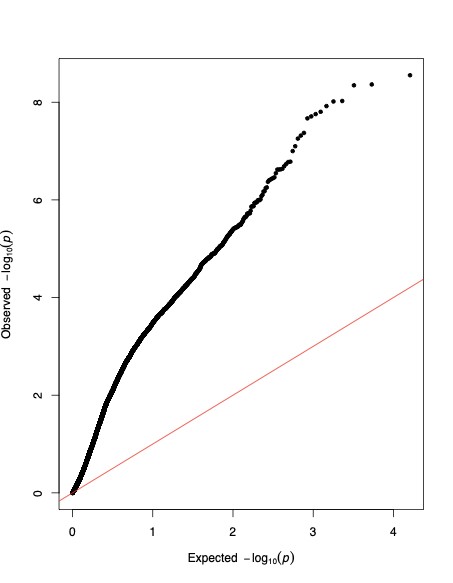


**Supplementary Figure 1. Q-Q plot for Age.** Probability plot was plotted that shows the expected versus the observed trend in the sample set. Drawing a correlation between the given sample set and normal distribution, the plot proves the strong association with age since we could see an excess of smaller p-values than expected.


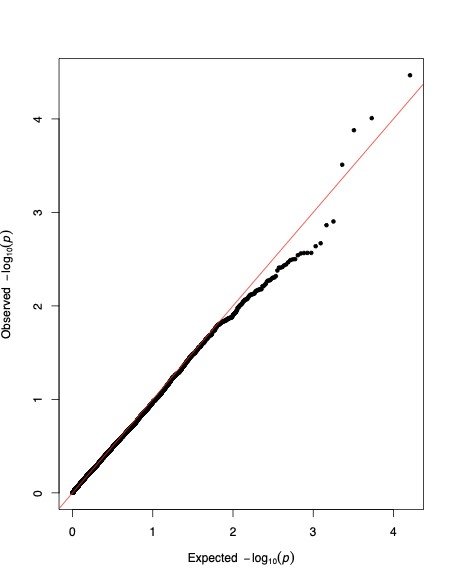


**Supplementary Figure 2. Q-Q plot for Milk Production.** Probability plot was plotted that shows the expected versus the observed trend in the sample set. Drawing a correlation between the given sample set and normal distribution for the milk production trait, the plot does not show much deviation indicating a correlation (if not strong) of epigenetics with milk production.

# DCC

−

l

o

g

10

(

*p*

)


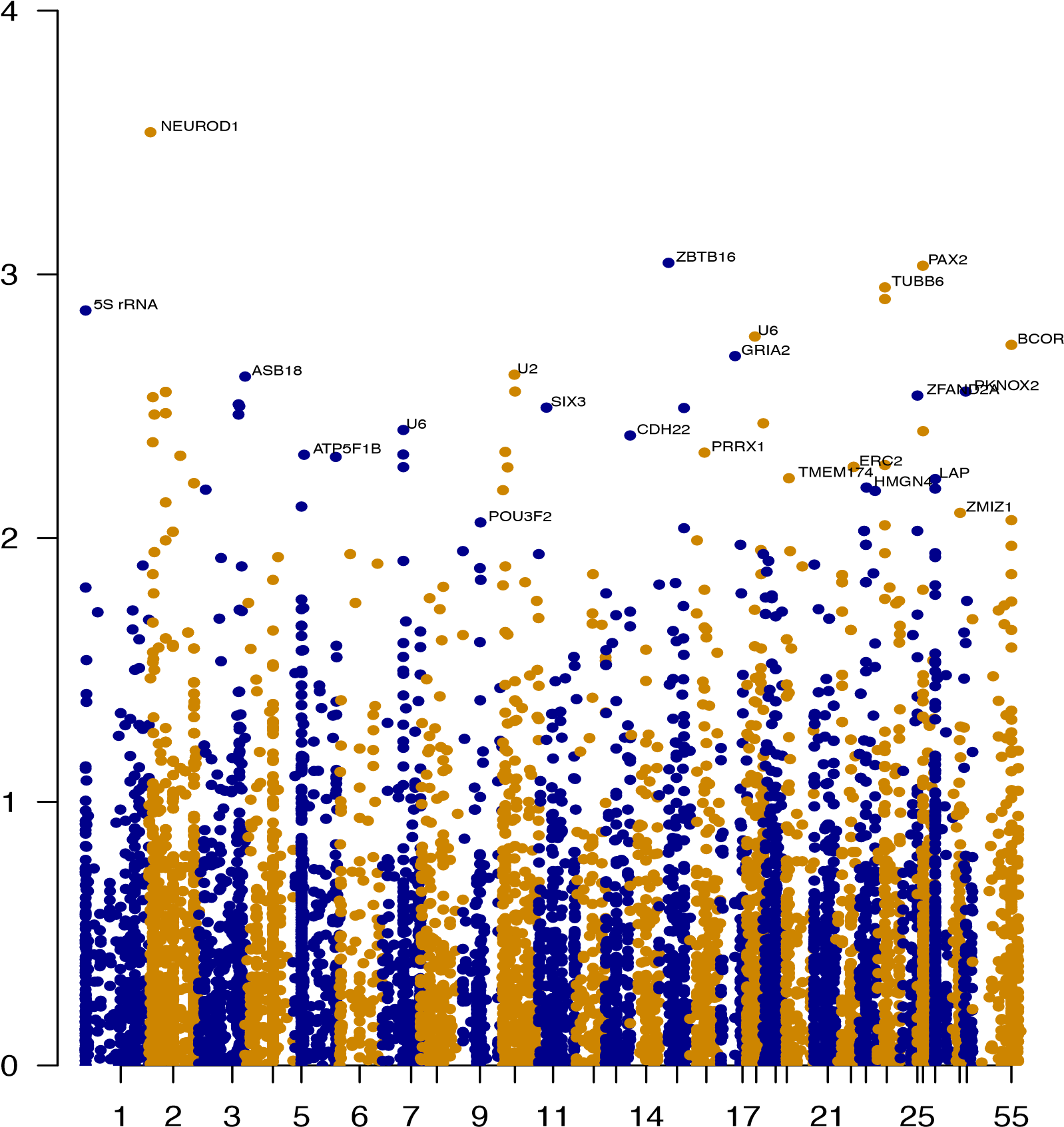


Chromosome

**Supplementary Figure 3. Epigenome-wide association results for DNAm DCC.** Manhattan plot was plotted presenting epigenome-wide association results for the days carried calf trait. Associations between single nucleotide polymorphisms and DCC have been plotted. CpG sites are plotted on the x-axis as per the chromosomal position and y-axis is -log_10_(p) for the trait of study which here is days carried calf; indicating higher the levels, stronger the association. There was no strong association observed between the trait of study and DNA methylation at different CpGs.

## Number of Lactations

−

l

o

g

10

(

*p*

)


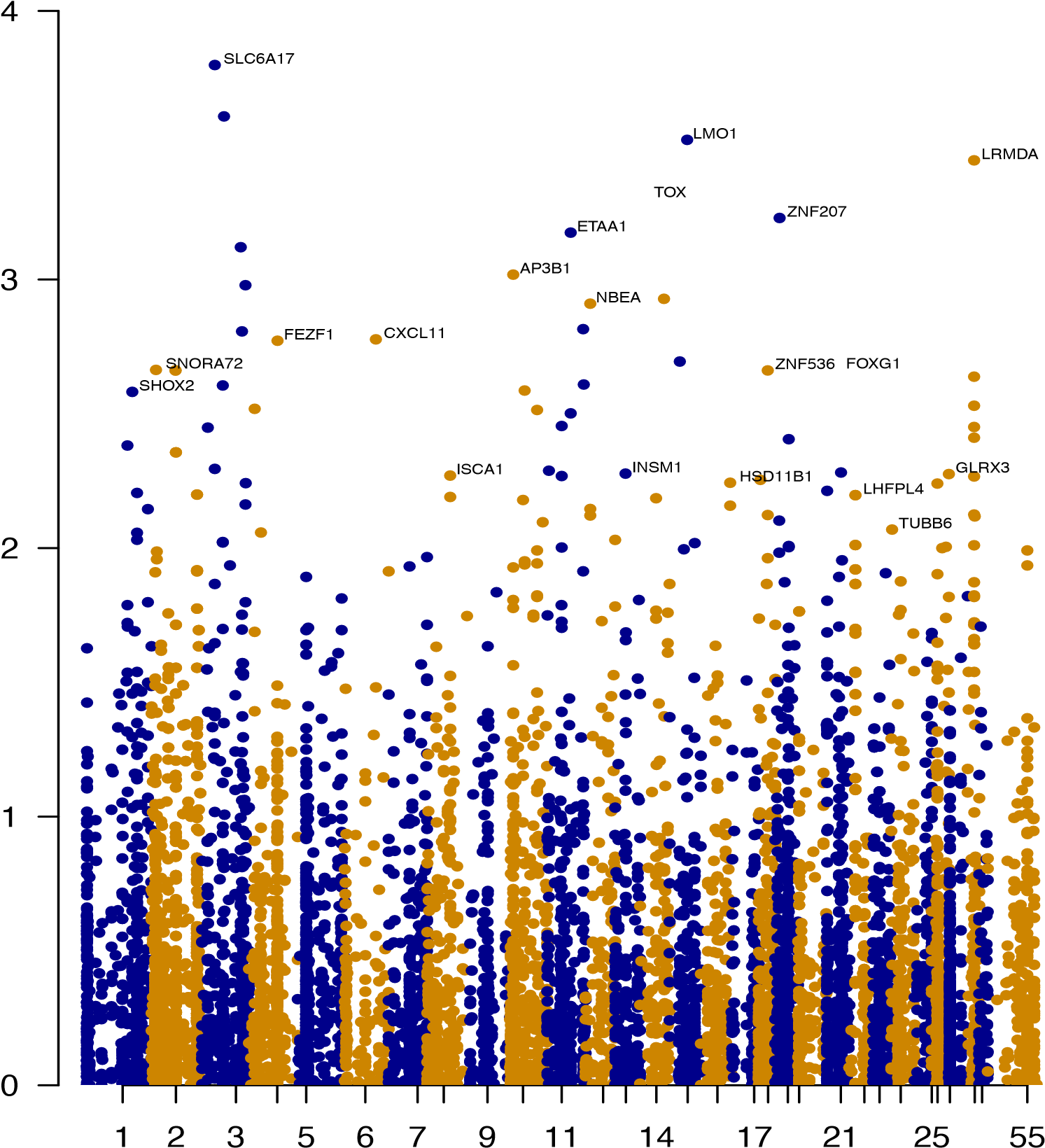


Chromosome

**Supplementary Figure 4. Epigenome-wide association results for DNAm Number of Lactations.** Manhattan plot was plotted presenting epigenome-wide association results for the number of lactations trait. Associations between single nucleotide polymorphisms and number of lactations have been plotted. CpG sites are plotted on the x-axis as per the chromosomal position and y-axis is -log_10_(p) for the trait of study which here is number of lactations; indicating higher the levels, stronger the association. There was no strong association observed between the trait of study and DNA methylation at different CpGs.

**Reproductive Status**

−

l

o

g

10

(

*p*

)


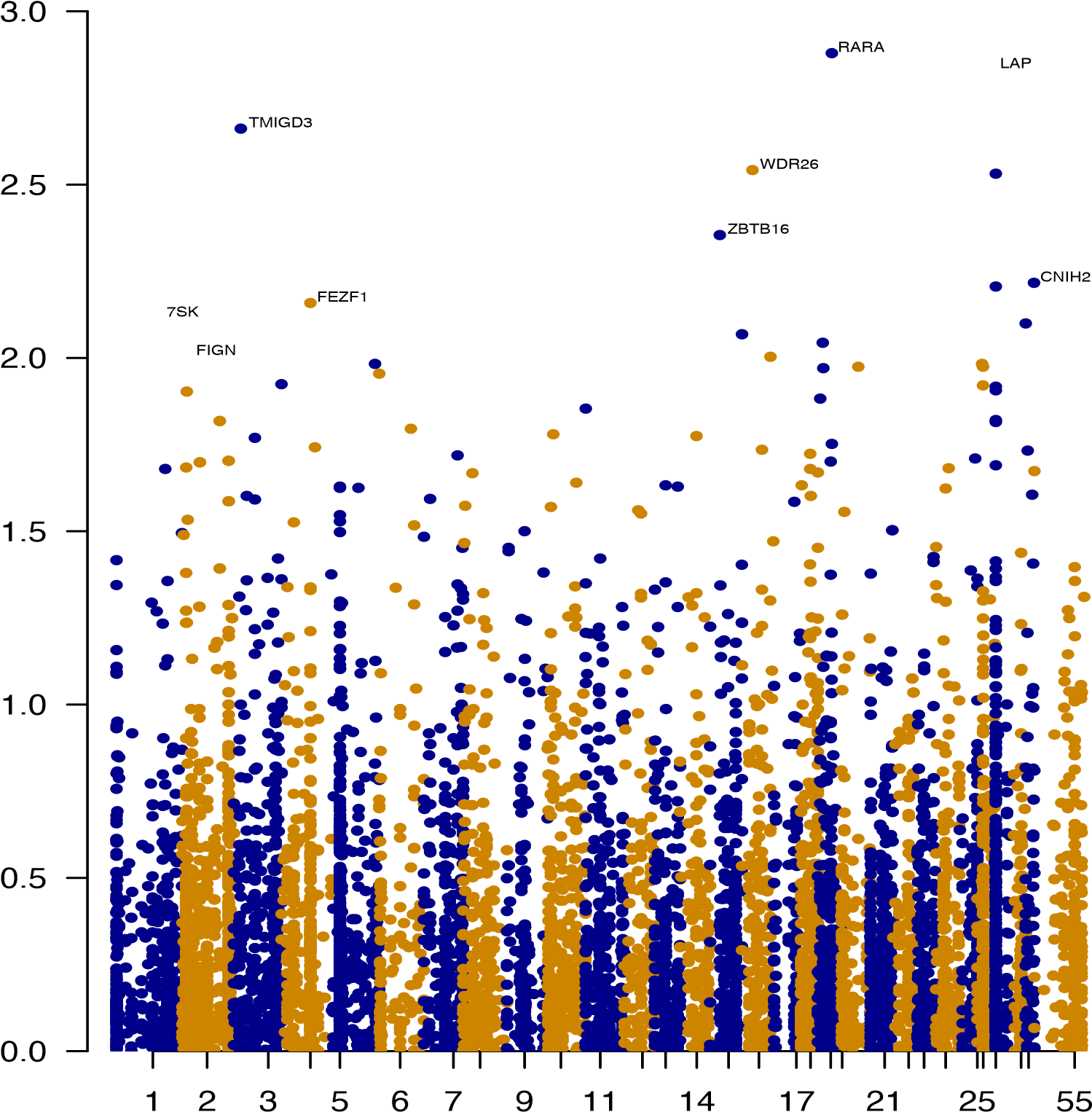


Chromosome

**Supplementary Figure 5. Epigenome-wide association results for DNAm Reproductive Status.** Manhattan plot was plotted presenting epigenome-wide association results for the reproductive status trait. Associations between single nucleotide polymorphisms and reproductive status have been plotted. CpG sites are plotted on the x-axis as per the chromosomal position and y-axis is -log_10_(p) for the trait of study which here is reproductive status; indicating higher the levels, stronger the association. There was no strong association observed between the trait of study and DNA methylation at different CpGs.
